# Neurologic adverse events in patients with relapsed/refractory acute lymphoblastic leukemia treated with blinatumomab: management and mitigating factors

**DOI:** 10.1007/s00277-018-3497-0

**Published:** 2018-09-20

**Authors:** Anthony S. Stein, Gary Schiller, Ramsis Benjamin, Catherine Jia, Alicia Zhang, Min Zhu, Zachary Zimmerman, Max S. Topp

**Affiliations:** 1 Department of Hematology/Hematopoietic Cell Transplantation, Gehr Leukemia Center, City of Hope National Medical Center, 1500 E. Duarte Road, Duarte, CA 91010-3000 USA; 2 David Geffen School of Medicine at UCLA, Los Angeles, CA USA; 3 Amgen Inc., South San Francisco, CA USA; 4 Amgen Inc., Thousand Oaks, CA USA; 5 Medizinische Klinik und Poliklinik II, Universitätsklinikum Würzburg, Würzburg, Germany

**Keywords:** Blinatumomab, BiTE®, Neurologic events, Adverse event, Exposure, Risk factor

## Abstract

Neurologic events (NEs) have been reported during treatment with blinatumomab, a bispecific T cell engager (BiTE®) construct. We evaluated the occurrence, severity, and management of NEs; the relationship between NEs and blinatumomab dose; and the potential clinical risk factors in an open-label, single-arm, phase 2 study (*N* = 189). Patients had Philadelphia chromosome–negative, relapsed/refractory acute lymphoblastic leukemia (ALL) and ≥ 10% bone marrow blasts. The relationship between blinatumomab exposure and NE incidence and severity was assessed. Clinical risk factors for NEs were assessed in a post hoc multivariate analysis. Overall, 98 patients (52%) experienced NEs: most frequently, dizziness, tremor, confusional state, and encephalopathy. NEs occurred predominantly during cycle 1 (median onset, 9 days) and were usually grades 1 or 2. Grade ≥ 3 NEs (13–17% incidence), serious NEs (16–19% incidence), and recurring NEs were managed with infusion interruptions or dexamethasone treatment. The incidence of NEs increased with increasing blinatumomab exposure at a given dose, but exposure appeared unrelated to NE severity. NEs were more frequent in patients ≥ 65 years than < 65 years (72 vs 49%). In a multivariate analysis, race other than white (hazard ratio [HR], 2.11; *P* = 0.009), > 2 prior salvage therapies (HR, 2.48; *P* = 0.006), and prior NEs (HR, 1.65; *P* = 0.020) were risk factors for time to first on-study NE. Although the mechanism underlying NEs associated with blinatumomab treatment in patients with relapsed/refractory ALL remains unclear, NEs tended to occur early during treatment and were often resolved by interrupting treatment and with dexamethasone. Additional research is warranted to investigate the risk factors for NEs.

**Electronic supplementary material:**

The online version of this article (10.1007/s00277-018-3497-0) contains supplementary material, which is available to authorized users.

## Introduction

Adult patients with B-precursor acute lymphoblastic leukemia (ALL) often achieve complete remissions (CRs) with conventional first-line treatment [1–3]. However, relapse rates are high despite allogeneic hematopoietic stem cell transplantation (alloHSCT) in high-risk patients [1]. Those who relapse, or are refractory to initial treatment, have a poor prognosis, with the likelihood of achieving a subsequent CR declining with each relapse [2, 3]. Novel treatment strategies employing immunotherapy agents are one means of addressing medical need for these patients. Blinatumomab is a 54-kDa bispecific T cell engager (BiTE®) antibody construct comprising CD19- and CD3-binding regions that link CD3-positive T lymphocytes with CD19-positive B cells, resulting in the serial lysis of target cells [4]. In a phase 2 study of blinatumomab (*N* = 189), 43% of adult patients with relapsed/refractory ALL achieved CR or CR with partial hematologic recovery of peripheral blood cells (CRh), allowing 40% of patients in remission to undergo alloHSCT [5]. In a recent randomized phase 3 study, blinatumomab significantly prolonged overall survival of adult patients with relapsed/refractory ALL compared with existing chemotherapy [6].

Blinatumomab treatment is associated with certain adverse events (AEs), such as pyrexia, fatigue, and headache, along with less frequent AEs like cytokine release syndrome (CRS), consistent with its effect on T cell function. Clinical studies exploring blinatumomab (60 μg/m^2^/day or 112 μg/day) in adult patients with ALL or non-Hodgkin lymphoma (NHL) have shown that treatment is also regularly associated with neurologic events (NEs), which represent the most frequent reasons for dose interruptions and discontinuations [4, 5, 7–9]. Given the nature of such events, their management requirements, and their impact on blinatumomab infusion, NEs represent a unique challenge during blinatumomab treatment.

We describe the occurrence patterns of NEs, their management, and the associated clinical risk factors in adult patients (*n* = 189) with relapsed/refractory ALL who received treatment with blinatumomab in a large phase 2 study. The primary analysis of this study is reported elsewhere [5].

## Methods

### Patients

Detailed eligibility requirements have been described previously; the protocol was approved by each center’s institutional review board or ethics committee, and all patients provided written informed consent [5]. Briefly, adults (≥ 18 years) with Philadelphia chromosome-negative B-precursor ALL whose disease was refractory to standard chemotherapy or had relapsed (first salvage with remission duration ≤ 12 months, or second or greater salvage) and who had ≥ 10% bone marrow blasts were eligible. Patients with alloHSCT < 3 months before starting blinatumomab and those with active ALL in the central nervous system (CNS) or with clinically relevant CNS pathology (e.g., epilepsy, seizure, paresis, aphasia, stroke, severe brain injuries, dementia, Parkinson’s disease, cerebellar disease, organic brain syndrome, or psychosis) were excluded from the study. However, enrollment of patients with a history of NEs was permitted at the investigator’s discretion if the NE was fully resolved at the time of screening.

### Study design and treatment

This single-arm, open-label, phase 2 study (*n* = 189) has been described in detail elsewhere [5]. The study followed a Simon two-stage design, with a third stage. A study amendment in June 2013 allowed enrollment of an additional evaluation cohort to examine neurologic symptoms and putative prognostic factors for NEs. In cycle 1, blinatumomab was administered at 9 μg/day on days 1 to 7 and then at 28 μg/day from days 8 to 29 via continuous intravenous infusion (IV). In the following cycles, blinatumomab was administered at 28 μg/day in 4-week cycles. There were two treatment-free weeks between cycles. Based on pharmacokinetic observations in previous studies, there was no adjustment of dose for body size [10]. Prephase dexamethasone treatment in patients with high tumor burden to mitigate risk of severe CRS and dexamethasone premedication before each cycle and dose step (cycle 1 only) to control infusion reactions was required [5]. Patients who achieved CR or CRh within the first two infusion cycles and those with hematologic relapse during follow-up could receive up to three additional cycles of treatment.

Blinatumomab infusion was stopped immediately in case of grade 3 NEs or serious NEs. If the NE returned to grade ≤ 1, blinatumomab could be restarted at 9 μg/day (dose escalation was not permitted) after a 2-week treatment-free interval. Before restarting blinatumomab, patients were premedicated with dexamethasone (and prophylactic anticonvulsant, if applicable). Blinatumomab treatment was permanently discontinued in case of grade 4 NEs, grade 3 NEs requiring infusion interruption at a dose of 9 μg/day, NEs requiring > 1 week to resolve to grade ≤ 1, or if a patient experienced more than one seizure.

### Assessments

AEs, including NEs, occurring during the core study (*n* = 189; from treatment start until 30 days after last treatment or before HSCT) were recorded and graded per the National Cancer Institute Common Terminology Criteria for Adverse Events (CTCAE), version 4.0 [11]. NEs were defined as previously reported [5]. Extended neurologic examinations assessing level of consciousness, orientation, vision, motor and sensory function, reflexes, muscle tone, coordination, and neuropsychological findings (e.g., speech, cognition, emotion) were performed at screening, at the start of each treatment cycle, at the end-of-core-study visit, and at the investigators’ discretion. NEs occurring during the core study or between the first infusion of the first retreatment cycle and 30 days after the last retreatment infusion are reported. Events that began before infusion start and worsened later were considered NEs.

In the additional evaluation cohort (*n* = 36), the following supplementary assessments were performed: extended neurologic examinations in cycle 1, including after the dose step, and daily when grade ≥ 2 NEs occurred (until resolution to grade ≤ 1 and once after resolution); a cranial magnetic resonance imaging (MRI) scan at screening, the end of the core study, grade ≥ 3 NE occurrence, and before retreatment after infusion interruption.

Blinatumomab concentrations in serum following continuous IV infusion were determined using a validated bioassay with a lower limit of quantitation of 50 pg/mL [4]. Samples were collected before IV infusion, on day 3 during infusion, on day 8 (before dose increase to 28 μg/day in cycle 1 and during 28 μg/day in cycle 2), and on days 15, 22, and 29 during IV infusion in cycles 1 and 2.

### Statistical analyses

The primary study endpoint was the rate of CR/CRh within the first two treatment cycles. Overall incidence and severity of AEs was a secondary endpoint. Time to onset and duration of NEs were summarized using descriptive statistics for patients who had at least one NE. Kaplan-Meier estimates and 95% confidence intervals were used to summarize data for all patients. Exploratory analyses using Cox proportional hazard models assessed the characteristics associated with time to the first occurrence of a NE. A stepwise regression method was used to construct a multivariate model, using 0.1 as inclusion and exclusion criteria for prespecified baseline characteristics. All data refer to the original evaluation cohort (*n* = 189) except where noted. No combined analysis of the two cohorts was performed.

## Results

### Patients

The study enrolled and treated 189 patients [5] before the protocol amendment allowed recruitment into the additional evaluation cohort (*n* = 36; Online Resource 1). Table 1 summarizes baseline demographics for patients who did and did not experience NEs during the study. Patients who developed NEs generally had a trend towards higher bone marrow blast count, had received multiple lines of prior therapy and a history of prior NEs, and were more likely to have received prephase dexamethasone. In both groups, the proportion of patients who had received prior CNS-directed therapy for ALL involvement was 15%. Although previous CNS pathology was an exclusion criterion, 66 patients (35%) had prior NEs (Online Resource 2) but were permitted to enroll because their events had fully resolved at the time of screening (see the “Methods” section). Two patients had prior seizures; one patient had prior posterior reversible encephalopathy syndrome.

**Table 1:** Baseline demographics and disease for patients with or without NEs on study

	Original evaluation cohort (*N* = 189)	
Characteristic	Patients with NEs (*n* = 98)	Patients without NEs (*n* = 91)
Median (range) age, year	41 (19–79)	34 (18–72)
Age < 65 year, *n* (%)	80 (82)	84 (92)
Age ≥ 65 year, *n* (%)	18 (18)	7 (8)
Sex, *n* (%)		
Men	59 (60)	60 (66)
Women	39 (40)	31 (34)
Race or ethnic group, *n* (%)		
White	71 (72)	74 (81)
Other^a^	16 (16)	8 (9)
Missing	11 (11)	9 (10)
Primary refractory, *n* (%)		
No	91 (93)	82 (90)
Yes^b^	7 (7)	9 (10)
Prior allogeneic HSCT, *n* (%)		
0	63 (64)	62 (68)
1	30 (31)	24 (26)
2 or 3	5 (5)	5 (5)
Prior salvage treatment, *n* (%)		
0	21 (21)	17 (19)
1	40 (41)	37 (41)
2	14 (14)	28 (31)
> 2	23 (23)	9 (10)
Bone marrow blast count per central read, *n* (%)		
< 50%	35 (36)	24 (26)
≥ 50%	63 (64)	67 (74)
Prephase dexamethasone administration, *n* (%)		
No	29 (30)	30 (33)
Yes	69 (70)	61 (67)
Prior history of NEs, *n* (%)		
No	55 (56)	68 (75)
Yes	43 (44)	23 (25)
Cranial radiation for CNS ALL, *n* (%)	15 (15)	14 (15)

*NE* neurologic event, *HSCT* hematopoietic stem cell transplantation

^a^Black (*n* = 7), Asian (*n* = 6), American Indian or Alaska Native (*n* = 1), Native Hawaiian or other Pacific Islander (*n* = 1), and other (*n* = 9). Race was not recorded for patients from France and one additional patient for privacy reasons

^b^Prior salvage therapies: none (*n* = 4), one (*n* = 7), two (*n* = 3), three or more (*n* = 2)

### NE incidence and timing

The incidence of NEs among the 189 patients and the 36 patients in the additional evaluation cohort was generally similar, including serious and grade ≥ 3 treatment-related NEs (Table 2). Overall, 98 (52%) patients in the original cohort and 15 (42%) in the additional evaluation cohort had NEs. The nature of the most frequently occurring NEs was also consistent between the two groups and included tremor, dizziness, confusional state, and encephalopathy (Online Resource 3) [5]. Four of the 189 patients had grade 4 NEs (encephalopathy, ataxia, convulsion, and febrile delirium). There were no grade 4 NEs in the additional evaluation cohort; however, two fatal NEs occurred after treatment had stopped. One patient with a history of ALL with CNS and testis involvement, prior treatment with total body irradiation, and approximately 10 intrathecal therapies for CNS died after developing encephalopathy and concurrent pneumonia during the treatment-free interval in cycle 1. The fatal event was considered related to blinatumomab and intense prestudy treatment. One patient who had multiple prior medical conditions stopped treatment on the 11th day after starting blinatumomab infusions in cycle 1 because of tachycardia. Three days later, the patient developed grade 4 metabolic encephalopathy and died after rapid progression. The investigator did not consider the metabolic encephalopathy related to blinatumomab treatment.

**Table 2:** Overview of NE incidence during the study

Patients with NEs, *n* (%)	Original evaluation cohort (*N* = 189)	Additional evaluation cohort (*N* = 36)
Any NE	98 (52)	15 (42)
Grade ≥ 3 NEs	24 (13)	6 (17)
Grade 3	20 (11)	4 (11)
Grade 4	4 (2)	0
Grade 5	0	2 (6)
Serious NEs	31 (16)	7 (19)
Any treatment-related NE	66 (35)	11 (31)
Treatment-related grade ≥ 3 NEs	22 (12)	4 (11)
Grade 3	18 (10)	3 (8)
Grade 4	4 (2)	0
Grade 5	0	1 (3)
Serious treatment-related NEs	27 (14)	5 (14)
NEs leading to dose interruption	29 (15)	5 (14)
NEs leading to permanent discontinuation	9 (5)	3 (8)

*NE* neurologic event

The rate of NEs of any grade and of grade ≥ 3 by cycle was highest in the first cycle (Table 3). The median time to onset of NEs of any grade was 9.0 days (*n* = 98), with median duration of 5 days; for NEs of grade ≥ 3, the onset was longer (16.5 days; *n* = 24) and duration was shorter (3 days). The median time to event resolution was shorter for patients who experienced NEs of grade ≥ 3 than for those with NEs of any grade (Table 3).

**Table 3:** Timing, duration, and incidence of NEs by treatment cycle

	Original evaluation cohort (*N* = 189)			
*Time to first onset and duration of NE resolution*				
	Median, days (IQR)	Range, days		
Time to first event^a^ (*n* = 98)	9.0 (4.0–20.0)	1–171		
Time to first grade ≥ 3 event^a^ (*n* = 24)	16.5 (10.0–51.5)	4–172		
*NE Incidence by blinatumomab treatment cycle*				
		Event Incidence		
Cycle number (number of patients who entered the cycle)	Patients with NEs *n* (%)	NE events *n*	Grade ≥ 2 *n* (%)	Grade ≥ 3 *n* (%)
Cycle 1^b^ (*n* = 189)	85 (45)	179	155 (82)	24 (13)
Cycle 2 (*n* = 98)	26 (27)	42	35 (36)	7 (7)
Cycle 3 (*n* = 43)	8 (19)	13	9 (21)	4 (9)
Cycle 4 (*n* = 22)	1 (5)	1	0	1 (5)
Cycle 5 (*n* = 12)	2 (17)	2	2 (17)	0
*NE duration*	Median, days (IQR)		Range, days	
Duration of NEs of any severity that were later resolved (*n* = 194 events resolved^a^	5.0 (2.0–14.0)		1–169	
Duration of grade ≥ 3 NEs that were later resolved (*n* = 29 events resolved)^a^	3.0 (2.0–5.0)		1–21	

*IQR* interquartile range, *NE* neurologic event

^a^Descriptive statistic; includes only those patients who experienced an NE

^b^Including 2-week treatment-free period

### Blinatumomab exposure

The incidence of NEs appeared to be associated with blinatumomab concentration at a given dose. In cycle 1, the number of patients with NEs increased with an increase in blinatumomab concentration at doses of 9 and 28 μg/day, respectively (Table 4). There was no apparent difference in blinatumomab steady-state concentrations (C_ss_) between patients experiencing NEs of different severity: mean (SD) C_ss_ was 711 pg/mL (541) and 635 pg/mL (279) at 28 μg/day for grade ≤ 2 (*n* = 62) and grade ≥ 3 NEs (*n* = 17), respectively.

**Table 4:** First Incidence of NEs by Blinatumomab C_ss_ in Cycle 1

	1st C_ss_ quartile	2nd C_ss_ quartile	3rd C_ss_ quartile	4th C_ss_ quartile
*Treatment during the first 7 days (9* μg*/day)*				
Median (range) C_ss_, pg/mL	68 (51–80)	109 (83–132)	147 (132–206)	387 (214–1782)
Number of patients with NEs, *n*/*N* (%)	5/21 (24)	3/21 (14)	7/21 (33)	10/22 (45)
*Treatment after 7 days (28* μg*/day)*				
Median (range) C_ss_, pg/mL	175 (57–264)	398 (266–494)	623 (494–815)	982 (821–2838)
Number of patients with NEs, *n*/*N* (%)	9/34 (26)	12/34 (35)	16/33 (48)	17/34 (50)

*C*_*ss*_ steady-state concentration, *NE* neurologic event

*n* the number of patients who had the first incidence of NE at any grade and quantifiable C_ss_ values, *N* the total number of patients who had quantifiable C_ss_

Across all cycles, total exposure (i.e., total dose received)-adjusted incidence rates for grade 1/2 and grade 3/4 NEs were 15.5 and 2.9 events per 100 weeks, respectively. When adjusting for total exposure, the incidence of NEs of any grade was similar between the two dosing groups. The incidence of serious NEs, such as tremor, confusional state, ataxia, and convulsion, but not encephalopathy, was higher with a dose of 28 μg/day (Table 5); however, this confounds with longer treatment at 28 μg/day, which was administered for all remaining doses after day 7.

**Table 5:** Incidence and exposure-adjusted event rate of NEs by dose

	Blinatumomab 9 μg/day (*n* = 189)		Blinatumomab 28 μg/day (*n* = 178)	
	Total crude exposure, 281.3 weeks		Total crude exposure, 1017.0 weeks	
Events^a^	Any NE *n* (%)	Serious NE *n* (%)	Any NE *n* (%)	Serious NE *n* (%)
Any NE^b^	58 (20.6)	4 (1.4)	179 (17.6)	35 (3.4)
Tremor	15 (5.3)	0	32 (3.1)	5 (0.5)
Dizziness	9 (3.2)	0	20 (2.0)	1 (0.1)
Paresthesia	7 (2.5)	1 (0.4)	0	0
Confusional state	5 (1.8)	0	11 (1.1)	5 (0.5)
Encephalopathy	3 (1.1)	2 (0.7)	7 (0.7)	3 (0.3)
Somnolence	3 (1.1)	0	10 (1.0)	0
Ataxia	2 (0.7)	0	7 (0.7)	3 (0.3)
Mental status changes	2 (0.7)	0	6 (0.6)	1 (0.1)
Lethargy	2 (0.7)	0	3 (0.3)	0
Aphasia	1 (0.4)	1 (0.4)	6 (0.6)	1 (0.1)
Dysarthria	1 (0.4)	0	6 (0.6)	1 (0.1)
Hypoesthesia	1 (0.4)	0	5 (0.5)	0
Neurotoxicity^c^	1 (0.4)	0	4 (0.4)	3 (0.3)
Nervous system disorder	1 (0.4)	0	2 (0.2)	1 (0.1)
Disorientation	0	0	8 (0.8)	0
Convulsion	0	0	4 (0.4)	2 (0.2)
Dysesthesia	0	0	4 (0.4)	2 (0.2)
Memory impairment	0	0	4 (0.4)	0
Cognitive disorder	0	0	3 (0.3)	3 (0.3)
Amnesia	0	0	2 (0.2)	0
Bradyphrenia	0	0	2 (0.2)	0
Delirium	0	0	2 (0.2)	0
Hyperreflexia	0	0	2 (0.2)	0
Nystagmus	0	0	2 (0.2)	0
Reflexes abnormal	0	0	2 (0.2)	0
Restless legs syndrome	0	0	2 (0.2)	0

*NE* neurologic event

^a^Event incidence rate per 100 weeks of exposure

^b^AEs of any grade reported in ≥ 2 patients in either group

^c^Event reported by the investigators without further specification

### Management, recurrence, and resolution of NEs

NEs of grade 3 and serious NEs were successfully managed with infusion interruptions and dexamethasone treatment of at least 3 × 8 mg/day with stepwise dose reduction over 4 days, as well as prophylactic anticonvulsant if the NE was a seizure. Overall, 29 (15%) patients had temporary interruptions or discontinuations of blinatumomab treatment, and 9 (5%) permanently discontinued blinatumomab due to NEs. Treatment interruption did not necessarily prevent subsequent remission [5]. Four patients experienced seizures on study and received interventional levetiracetam; one patient stopped blinatumomab permanently due to NEs.

### Baseline risk factors for NEs

We previously observed in an analysis of two phase 2 studies that relapsed/refractory ALL patients aged ≥ 65 years had a greater incidence of NEs (any grade and grade ≥ 3) than patients < 65 years following treatment with blinatumomab monotherapy [12]. In the present study, patients ≥ 65 years, compared with < 65 years, had a significantly higher incidence of both prior neurologic disorders (52 vs 32%; *P* = 0.054) and on-study NEs (72 vs 49%; *P* = 0.030) and were more likely to have treatment interruptions (36 vs 12%; *P* = 0.002) and discontinuations (12 vs 4%; *P* = 0.068) because of NEs. Patients ≥ 65 years presented primarily with dizziness (32%), confusional state, encephalopathy, and tremor (16% each) and aphasia, ataxia, and somnolence (12% each). Except for tremor, these NEs also occurred more frequently in older than in younger patients. Dizziness, confusional state, encephalopathy, and ataxia occurred more frequently in patients with a history of previous NE (Table 6).

**Table 6:** Incidence of NEs occurring in ≥ 2% of patients with or without prior NEs

Patients with events	Patients with prior NEs (*n* = 66)	Patients without prior NEs (*n* = 123)
Any NE, *n* (%)	43 (65)	55 (45)
Dizziness	16 (24)	10 (8)
Tremor	12 (18)	21 (17)
Confusional state	10 (15)	4 (3)
Encephalopathy	6 (9)	4 (3)
Ataxia	6 (9)	3 (2)
Neurotoxicity	4 (6)	1 (1)
Lethargy	4 (6)	1 (1)
Convulsion	4 (6)	0
Aphasia	3 (5)	4 (3)
Dysarthria	3 (5)	3 (2)
Somnolence	2 (3)	7 (6)
Paresthesia	2 (3)	5 (4)
Disorientation	2 (3)	5 (4)
Mental status changes	2 (3)	5 (4)
Hypoesthesia	2 (3)	4 (3)
Memory impairment	2 (3)	1 (1)
Dysesthesia	2 (3)	1 (1)
Delirium	2 (3)	0
Reflexes abnormal	2 (3)	0
Amnesia	1 (2)	1 (1)
Depressed level of consciousness	1 (2)	0
Febrile delirium	1 (2)	0
Hemiparesis	1 (2)	1 (1)
Hyperreflexia	1 (2)	1 (1)
Facial nerve disorder	1 (2)	0
Leukoencephalopathy	1 (2)	0
Myoclonus	1 (2)	0
Postural dizziness	1 (2)	0
Restless legs syndrome	1 (2)	1 (1)
Bradyphrenia	0	2 (2)
Nervous system disorder	0	3 (2)
Cognitive disorder	0	3 (2)
Nystagmus	0	2 (2)

*NE* neurologic event

In a post hoc univariate analysis of baseline characteristics, patients with > 2 prior salvage therapies compared with those without prior salvage therapies (HR, 2.11; *P* = 0.014), those with prior NEs (HR, 1.63; *P* = 0.017), and those with race other than white (HR, 1.68; *P* = 0.061) were risk factors for time to first on-study NE (Online Resource 4). In a multivariate analysis, all three characteristics remained statistically significant as risk factors for time to first on-study NE: race other than white (HR, 2.11; *P* = 0.009), > 2 prior salvage therapies (HR, 2.48; *P* = 0.006), and prior NEs (HR, 1.65; *P* = 0.020).

There was no apparent association between the incidence of CRS and NEs. Fifty-eight percent of patients with CRS (*n* = 24) also had treatment-concurrent NEs, compared with 51% of patients without CRS and NEs (*n* = 165). Fourteen patients had both CRS and NEs, with CRS preceding the NE in all but one case.

## Discussion

Treatment with the CD19-targeted immunotherapy blinatumomab is associated with NEs [4, 5, 7–9]. In this analysis of a large phase 2 study of blinatumomab in adults with relapsed/refractory ALL [5], the most frequent on-study NEs (e.g., tremor, dizziness, confusional state, and encephalopathy) have been reported consistently across blinatumomab studies, including the recent phase 3 TOWER study in patients with ALL [4–9]. Notably, most NEs were grade 1 or 2 and occurred early in cycle 1 when patients received the initial blinatumomab dose step in the hospital.

The NE incidence appeared to increase with increasing blinatumomab exposure at a given dose but factors other than drug concentration may have affected the results. In a recent publication, higher C_ss_ with blinatumomab at a dose of 9 μg/day was associated with a greater incidence of NE events in the univariate analysis but not in the multivariate analysis [13]. Other disease- or treatment-related factors, such as more than two prior salvage therapies, may have affected the occurrence of NEs. The present study showed that there was no difference in median exposure between patients experiencing grade ≤ 2 and grade ≥ 3 NEs, suggesting that blinatumomab concentrations may not be a key driver of NE severity.

NEs were successfully managed by blinatumomab dose interruptions or discontinuations in 29 patients. The occurrence of NEs did not appear to hinder achievement of remission. Of the 29 patients with treatment interruptions or discontinuations due to NEs, 10 achieved CR/CRh before interruption, and 8 achieved CR/CRh after restarting treatment [5]. Blinatumomab dose interruption with dexamethasone was also successful for the management in patients who experienced additional NEs after previously delaying blinatumomab due to NEs, as well as for more serious NEs, including seizures, encephalopathy, and aphasia. Although the protocol did not mandate primary seizure prophylaxis, it recommended secondary prophylaxis with phenytoin or levetiracetam, which was administered to all four patients who experienced seizures, allowing three of them to continue treatment. The data show that close monitoring with early appropriate intervention supported successful management of NEs with minimal impact on the treatment course for most of the patients.

Although previous analyses of phase 2 data have shown that patients aged ≥ 65 years with relapsed/refractory ALL had a greater incidence of NEs [12], the multivariate model in this study did not demonstrate older age alone as a significant risk factor for time to first on-study NE. Furthermore, the types of NEs seen more frequently in older patients were consistent with those expected in this population, and older patients were more likely to have treatment interruptions as a result. The results of the multivariate analysis suggest that prior history of neurologic disorders should be considered before initiation of blinatumomab treatment. However, given the small number of patients in the analysis and considering that such measures may simply reflect disease severity—healthier patients are less likely to experience NEs—these prognostic evaluations should be interpreted with caution.

Based on the blinatumomab mode of action, T cell activation and subsequent cytokine release are known to play a role in the development of certain blinatumomab-associated AEs, such as pyrexia, fatigue, headache, and CRS [14]. In contrast, the mechanism underlying the development of NEs is not yet understood but likely multifactorial. Tumor burden may not contribute to the development of NEs given the similarity in rates of grade 3/4 NEs in patients with minimal residual disease [15]. Similar NEs, such as encephalopathy and seizures, have been reported in patients receiving CD19-targeted chimeric antigen receptor (CAR) T cells, including severe and fatal events [16–18], suggesting a possible target dependence. Although more patients with NEs also had CRS, we did not find an association between NEs, CRS, and/or peak cytokine levels, especially interleukin (IL)-6, unlike recent reports with CD19 CAR T cells in which patients with severe neurotoxicity also developed CRS characterized by elevated IL-6 and interferon gamma [18]. In other studies of CAR T cells, CAR T cells were detected in the spinal fluid of nearly all patients regardless of the occurrence of NEs [17, 19]. B and T cells, as well as blinatumomab, have been detected in the spinal fluid of adult and pediatric patients treated with blinatumomab for ALL and NHL; however, these were independent of blood–CSF barrier integrity [20]. Expression of CD19 in the CNS has been investigated as a potential factor in the development of motor aphasia in patients receiving CD19 CAR T cells for hematologic malignancies [21]. Unfortunately, the MRI data in the current study were insufficient to investigate where pathologic changes in the CNS may have contributed to the development of NEs associated with blinatumomab. Further research in this area is required.

In this analysis of patients with relapsed/refractory ALL who received blinatumomab, NEs occurred early during treatment and could be managed with dose interruption or medication, even after recurrence. Increased exposure to blinatumomab and older age appeared to increase the incidence of certain NEs but not the severity. In a post hoc multivariate analysis, race, prior salvage therapy, and history of NEs were risk factors for time to first on-study NE. Unlike CAR T cells, peak interleukin levels and CRS do not appear to contribute to the development of NEs with blinatumomab treatment. Further investigation of the underlying mechanisms of NEs during blinatumomab treatment is warranted.

## Electronic supplementary material

ESM 1 (DOCX 124 kb)

## References

[1] Richards SM, Chopra R, Lazarus HM, Litzow MR, Buck G, Durrant IJ, Luger SM, Marks DI, Franklin IM, AK MM, Tallman MS, Rowe JM, Goldstone AH, Fielding AK, Medical Research Council of the United Kingdom Adult ALLWP, Eastern Cooperative Oncology G (2007). Outcome of 609 adults after relapse of acute lymphoblastic leukemia (ALL); an MRC UKALL12/ECOG 2993 study. Blood.

[2] Gokbuget N, Stanze D, Beck J, Diedrich H, Horst HA, Huttmann A, Kobbe G, Kreuzer KA, Leimer L, Reichle A, Schaich M, Schwartz S, Serve H, Starck M, Stelljes M, Stuhlmann R, Viardot A, Wendelin K, Freund M, Hoelzer D, German Multicenter Study Group for Adult Acute Lymphoblastic L (2012). Outcome of relapsed adult lymphoblastic leukemia depends on response to salvage chemotherapy, prognostic factors, and performance of stem cell transplantation. Blood.

[3] O'Brien S, Thomas D, Ravandi F, Faderl S, Cortes J, Borthakur G, Pierce S, Garcia-Manero G, Kantarjian HM (2008). Outcome of adults with acute lymphocytic leukemia after second salvage therapy. Cancer.

[4] Bargou R, Leo E, Zugmaier G, Klinger M, Goebeler M, Knop S, Noppeney R, Viardot A, Hess G, Schuler M, Einsele H, Brandl C, Wolf A, Kirchinger P, Klappers P, Schmidt M, Riethmuller G, Reinhardt C, Baeuerle PA, Kufer P (2008). Tumor regression in cancer patients by very low doses of a T cell-engaging antibody. Science.

[5] Topp MS, Gokbuget N, Stein AS, Zugmaier G, O'Brien S, Bargou RC, Dombret H, Fielding AK, Heffner L, Larson RA, Neumann S, Foa R, Litzow M, Ribera JM, Rambaldi A, Schiller G, Bruggemann M, Horst HA, Holland C, Jia C, Maniar T, Huber B, Nagorsen D, Forman SJ, Kantarjian HM (2015). Safety and activity of blinatumomab for adult patients with relapsed or refractory B-precursor acute lymphoblastic leukaemia: a multicentre, single-arm, phase 2 study. Lancet Oncol.

[6] Topp MS, Stein A, Gökbuget N, Fielding A, Schuh A, Maria Ribera Santasusana J, Wei J, Dombret H, Foà R, Bassan R, Arslan O, Sanz MA, Bergeron J, Demirkan F, Lech-Maranda E, Rambaldi A, Thomas X, Fleishman A, Nagorsen D, Holland C, Zimmerman Z, Kantarjian H (2016). Blinatumomab improved overall survival in patients with relapsed or refractory Philadelphia negative B-cell precursor acute lymphoblastic leukemia in a randomized, open-label phase 3 study (TOWER). Haematologica.

[7] Topp MS, Gokbuget N, Zugmaier G, Klappers P, Stelljes M, Neumann S, Viardot A, Marks R, Diedrich H, Faul C, Reichle A, Horst HA, Bruggemann M, Wessiepe D, Holland C, Alekar S, Mergen N, Einsele H, Hoelzer D, Bargou RC (2014). Phase II trial of the anti-CD19 bispecific T cell-engager blinatumomab shows hematologic and molecular remissions in patients with relapsed or refractory B-precursor acute lymphoblastic leukemia. J Clin Oncol.

[8] Goebeler ME, Knop S, Viardot A, Kufer P, Topp MS, Einsele H, Noppeney R, Hess G, Kallert S, Mackensen A, Rupertus K, Kanz L, Libicher M, Nagorsen D, Zugmaier G, Klinger M, Wolf A, Dorsch B, Quednau BD, Schmidt M, Scheele J, Baeuerle PA, Leo E, Bargou RC (2016). Bispecific T-cell engager (BiTE) antibody construct blinatumomab for the treatment of patients with relapsed/refractory non-Hodgkin lymphoma: final results from a phase I study. J Clin Oncol.

[9] Viardot A, Goebeler ME, Hess G, Neumann S, Pfreundschuh M, Adrian N, Zettl F, Libicher M, Sayehli C, Stieglmaier J, Zhang A, Nagorsen D, Bargou RC (2016). Phase 2 study of the bispecific T-cell engager (BiTE) antibody blinatumomab in relapsed/refractory diffuse large B-cell lymphoma. Blood.

[10] Zhu M, Wu B, Brandl C, Johnson J, Wolf A, Chow A, Doshi S (2016). Blinatumomab, a bispecific T-cell engager (BiTE(R)) for CD-19 targeted cancer immunotherapy: clinical pharmacology and its implications. Clin Pharmacokinet.

[11] National Cancer Institute (2014) NCI Common Terminology Criteria for Adverse Events (CTCAE) v4.0*.* Bethesda, MD,

[12] Kantarjian HM, Stein AS, Bargou RC, Grande Garcia C, Larson RA, Stelljes M, Gokbuget N, Zugmaier G, Benjamin JE, Zhang A, Jia C, Topp MS (2016). Blinatumomab treatment of older adults with relapsed/refractory B-precursor acute lymphoblastic leukemia: results from 2 phase 2 studies. Cancer.

[13] Zhu M, Kratzer A, Johnson J, Holland C, Brandl C, Singh I, Wolf A, Doshi S (2018). Blinatumomab pharmacodynamics and exposure-response relationships in relapsed/refractory acute lymphoblastic leukemia. J Clin Pharmacol.

[14] Barrett DM, Teachey DT, Grupp SA (2014). Toxicity management for patients receiving novel T-cell engaging therapies. Curr Opin Pediatr.

[15] Gokbuget N, Dombret H, Bonifacio M, Reichle A, Graux C, Faul C, Diedrich H, Topp MS, Bruggemann M, Horst HA, Havelange V, Stieglmaier J, Wessels H, Haddad V, Benjamin JE, Zugmaier G, Nagorsen D, Bargou RC (2018). Blinatumomab for minimal residual disease in adults with B-cell precursor acute lymphoblastic leukemia. Blood.

[16] Davila ML, Riviere I, Wang X, Bartido S, Park J, Curran K, Chung SS, Stefanski J, Borquez-Ojeda O, Olszewska M (2014). Efficacy and toxicity management of 19-28z CAR T cell therapy in B cell acute lymphoblastic leukemia. Sci Transl Med.

[17] Maude SL, Frey N, Shaw PA, Aplenc R, Barrett DM, Bunin NJ, Chew A, Gonzalez VE, Zheng Z, Lacey SF, Mahnke YD, Melenhorst JJ, Rheingold SR, Shen A, Teachey DT, Levine BL, June CH, Porter DL, Grupp SA (2014). Chimeric antigen receptor T cells for sustained remissions in leukemia. N Engl J Med.

[18] Turtle CJ, Hanafi LA, Berger C, Gooley TA, Cherian S, Hudecek M, Sommermeyer D, Melville K, Pender B, Budiarto TM, Robinson E, Steevens NN, Chaney C, Soma L, Chen X, Yeung C, Wood B, Li D, Cao J, Heimfeld S, Jensen MC, Riddell SR, Maloney DG (2016). CD19 CAR-T cells of defined CD4+:CD8+ composition in adult B cell ALL patients. J Clin Invest.

[19] Maude SL, Teachey DT, Porter DL, Grupp SA (2015). CD19-targeted chimeric antigen receptor T-cell therapy for acute lymphoblastic leukemia. Blood.

[20] Klinger M, Zugmaier G, Naegele V, Goebeler M, Brandl C, Bargou R, Kufer P (2016). Pathogenesis-based development of potential mitigation strategies for blinatumomab-associated neurologic events (NEs). Blood.

[21] Kranick S, Phan G, Kochenderfer JN, Rosenberg SA, Nath A (2014). Aphasia as a complication of CD19-targeted chimeric antigen receptor immunotherapy. Neurology.

