# Supplementary material for: Neurologic adverse events in patients with relapsed/refractory acute lymphoblastic leukemia treated with blinatumomab: management and mitigating factors
Source: Ann Hematol. 2018 Sep 20;98(1):159–67. doi: 10.1007/s00277-018-3497-0 (PMC6334725; doi:10.1007/s00277-018-3497-0)
Supplement: Supplementary file 1 — (DOCX 124 kb) [file 277_2018_3497_MOESM1_ESM.docx]

**Neurologic Adverse Events in Patients With Relapsed/Refractory Acute Lymphoblastic Leukemia Treated With Blinatumomab: Management and Mitigating Factors**

Anthony S. Stein, Gary Schiller, Ramsis Benjamin, Catherine Jia, Alicia Zhang, Min Zhu, Zachary Zimmerman, Max S. Topp

# ONLINE RESOURCES

Online Resource 1. **Patient Disposition in the Additional Evaluation Cohort**

The study was based on a Simon two-stage design with a third stage and an additional evaluation cohort that was added per protocol amendment. The primary analysis was conducted after the third enrollment stage and did not include the additional evaluation cohort. The core study was defined as the treatment period plus 30 days after the last administered blinatumomab dose, or before allogeneic hematopoietic stem cell transplantation or other treatment for acute lymphoblastic leukemia. MRI, magnetic resonance imaging.

Topp M et al *Lancet Oncology* 2015;16:57–66.


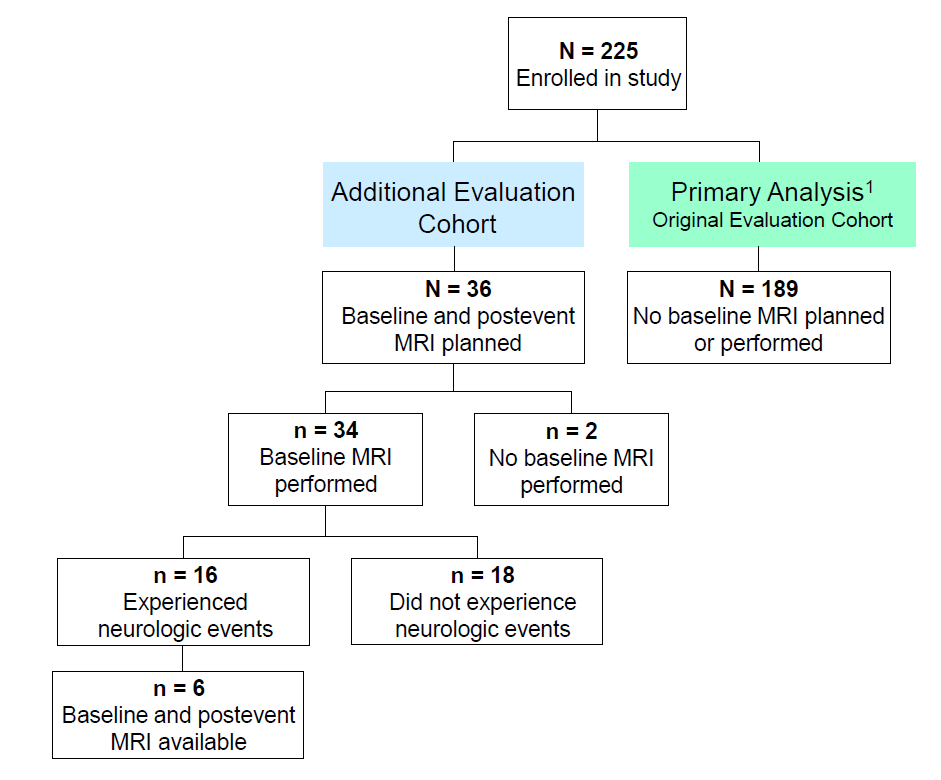


**Online Resource 2. Incidence of Neurologic Events Before Study Enrollment**

| **Patients With NEs** | **N (%)** |
| --- | --- |
| Any NE | 66 (35) |
| Dizziness | 11 (17) |
| Hypoesthesia | 7 (11) |
| Hyporeflexia | 6 (9) |
| Tremor | 6 (9) |
| Dysesthesia | 5 (8) |
| Paresthesia | 5 (8) |
| Memory impairment | 4 (6) |
| Drug abuse | 3 (5) |
| Neuralgia | 3 (5) |
| Balance disorder | 2 (3) |
| Confusional state | 2 (3) |
| Convulsion | 2 (3) |
| Hemiparesis | 2 (3) |
| Mental status changes | 2 (3) |
| Reflexes abnormal | 2 (3) |
| Restless legs syndrome | 2 (3) |
| Somnolence | 2 (3) |
| Tobacco abuse | 2 (3) |
| VIIth nerve paralysis | 2 (3) |
| Ageusia | 1 (2) |
| Alcohol abuse | 1 (2) |
| Alcoholism | 1 (2) |
| Areflexia | 1 (2) |
| Attention deficit/hyperactivity disorder | 1 (2) |
| Cerebellar syndrome | 1 (2) |
| Complex regional pain syndrome | 1 (2) |
| Dependence | 1 (2) |
| Disturbance in attention | 1 (2) |
| Dysgeusia | 1 (2) |
| Facial nerve disorder | 1 (2) |
| Facial paresis | 1 (2) |
| Morton’s neuralgia | 1 (2) |
| Nicotine dependence | 1 (2) |
| Nystagmus | 1 (2) |
| Paraplegia | 1 (2) |
| Postherpetic neuralgia | 1 (2) |
| Posterior reversible encephalopathy syndrome | 1 (2) |
| Postural dizziness | 1 (2) |
| Psychotic disorder | 1 (2) |
| Syncope | 1 (2) |
| Trigeminal nerve paresis | 1 (2) |

NE, neurologic event

Online Resource 3. Incidence of NEs by Worst Grade in the Additional Evaluation Cohort

|  | | **Additional Evaluation Cohort (N = 36)** | | | | | |
| --- | --- | --- | --- | --- | --- | --- | --- |
| **Patients with NEs, n (%)** | **All Grades*** | | **Grade 1** | **Grade 2** | **Grade 3** | **Grade 4** | **Grade 5** |
| Any NE | 15 (42) | | 3 (8) | 6 (17) | 4 (11) | 0 | 2 (6) |
| Convulsion | 3 (8) | | 1 (3) | 1 (3) | 1 (3) | 0 | 0 |
| Dizziness | 3 (8) | | 3 (8) | 0 | 0 | 0 | 0 |
| Tremor | 3 (8) | | 1 (3) | 2 (6) | 0 | 0 | 0 |
| Aphasia | 2 (6) | | 1 (3) | 0 | 1 (3) | 0 | 0 |
| Confusional state | 2 (6) | | 0 | 1 (3) | 1 (3) | 0 | 0 |
| Paresthesia | 2 (6) | | 1 (3) | 0 | 1 (3) | 0 | 0 |
| Somnolence | 2 (6) | | 1 (3) | 1 (3) | 0 | 0 | 0 |
| Disorientation | 1 (3) | | 0 | 0 | 1 (3) | 0 | 0 |
| Encephalopathy | 1 (3) | | 0 | 0 | 0 | 0 | 1 (3) |
| Hypoesthesia | 1 (3) | | 0 | 1 (3) | 0 | 0 | 0 |
| Hyporeflexia | 1 (3) | | 1 (3) | 0 | 0 | 0 | 0 |
| Lethargy | 1 (3) | | 1 (3) | 0 | 0 | 0 | 0 |
| Metabolic encephalopathy | 1 (3) | | 0 | 0 | 0 | 0 | 1 (3) |
| Neurological symptom | 1 (3) | | 0 | 0 | 1 (3) | 0 | 0 |
| Syncope | 1 (3) | | 0 | 1 (3) | 0 | 0 | 0 |

NE, neurologic event.

*Grades represent maximum severity

**Online Resource 4. Risk of Time to First On-Study NE by Baseline Variable**

| **Covariates** | **No. of Events/No. of Patients (%)** | **HR (95% CI)^†^** | ***P* Value** |
| --- | --- | --- | --- |
| *Univariate analysis* | | | |
| Age |  |  |  |
| <65 years* | 80/164 (49) |  |  |
| ≥65 years | 18/25 (72) | 1.60 (0.96–2.68) | 0.072 |
| Sex |  |  |  |
| Male* | 59/119 (50) |  |  |
| Female | 39/70 (56) | 1.15 (0.77–1.73) | 0.495 |
| Race |  |  |  |
| White* | 71/145 (49) |  |  |
| Other | 16/24 (67) | 1.68 (0.98–2.90) | 0.061 |
| Region |  |  |  |
| North America* | 48/94 (51) |  |  |
| Europe | 50/95 (53) | 1.21 (0.82–1.81) | 0.338 |
| Primary refractory |  |  |  |
| No* | 91/173 (53) |  |  |
| Yes | 7/16 (44) | 0.73 (0.34–1.58) | 0.428 |
| Prior allogeneic HSCT |  |  |  |
| 0* | 7/16 (44) |  |  |
| 1 | 48/84 (57) | 1.47 (0.66–3.25) | 0.343 |
| ≥2 | 8/25 (32) | 0.78 (0.28–2.15) | 0.632 |
| Prior salvage therapies |  |  |  |
| 0* | 21/38 (55) |  |  |
| 1 | 40/77 (52) | 0.89 (0.53–1.52) | 0.676 |
| 2 | 14/42 (33) | 0.56 (0.28–1.10) | 0.091 |
| >2 | 23/32 (72) | 2.11 (1.16–3.83) | 0.014 |
| Baseline central bone marrow |  |  |  |
| <50%* | 35/59 (59) |  |  |
| ≥50% | 63/130 (48) | 0.81 (0.54–1.23) | 0.328 |
| Prephase dexamethasone use |  |  |  |
| No* | 29/59 (49) |  |  |
| Yes | 69/130 (53) | 1.09 (0.70–1.68) | 0.710 |
| Prior NEs |  |  |  |
| No* | 55/123 (45) |  |  |
| Yes | 43/66 (65) | 1.63 (1.09–2.42) | 0.017 |
| Cranial radiation for CNS ALL |  |  |  |
| No* | 83/160 (52) |  |  |
| Yes | 15/29 (52) | 1.16 (0.67–2.01) | 0.603 |
| *Multivariate analysis* |  |  |  |
| Race |  |  |  |
| White* | 71/145 (49) |  |  |
| Other | 16/24 (67) | 2.11 (1.21–3.68) | 0.009 |
| Prior salvage therapies |  |  |  |
| 0* | 21/38 (55) |  |  |
| 1 | 40/77 (52) | 0.82 (0.46–1.48) | 0.510 |
| 2 | 14/42 (33) | 0.68 (0.33–1.38) | 0.284 |
| >2 | 23/32 (72) | 2.48 (1.30–4.71) | 0.0006 |
| Prior NEs |  |  |  |
| No* | 55/123 (45) |  |  |
| Yes | 43/66 (65) | 1.65 (1.08–2.53) | 0.020 |

ALL, acute lymphoblastic leukemia; CI, confidence interval; CNS, central nervous system; HR, hazard ratio; HSCT, hematopoietic stem cell transplant; NE, neurologic event.

*Reference group.

^†^HR is relative to the reference group; HR <1 indicates a lower risk of having an event in the test group than in the reference group.
